# Elevated METS-IR is associated with an increased risk of osteoarthritis: A cross-sectional NHANES-based study

**DOI:** 10.1097/MD.0000000000045279

**Published:** 2025-10-24

**Authors:** Junpeng Qiu, Muyuan Hou, Jiangfeng Lv, Yaoxin Ao, Yifei Liufu, Junxing Yang, Fangjun Xiao

**Affiliations:** a Department of Orthopaedics, Shenzhen Hospital (Futian) of Guangzhou University of Chinese Medicine, Shenzhen, China; b The Sixth Clinical Medical College, Guangzhou University of Chinese Medicine, Guangzhou, China.

**Keywords:** insulin resistance, metabolic syndrome, METS-IR, NHANES, osteoarthritis

## Abstract

Osteoarthritis (OA) is a prevalent chronic joint disease, metabolic abnormalities may play a key role in its development and progression. The metabolic score for insulin resistance (METS-IR) is an emerging index used to assess insulin resistance and metabolic dysfunction, but its relationship with OA remains unclear. This study utilized the National Health and Nutrition Examination Survey 2011 to 2018 database, including 6079 participants aged ≥20 years, to investigate the association between METS-IR and OA. OA was defined based on self-reported physician diagnoses. METS-IR was calculated using fasting glucose, triglycerides, HDL cholesterol, and body mass index (BMI). Multivariate logistic regression was employed to analyze the relationship between METS-IR and OA, while receiver operating characteristic (ROC) curves were used to evaluate the predictive efficacy of METS-IR for OA risk. Nonlinear associations were explored through smoothed curve fitting, and subgroup analyses were performed to assess potential interactions. Elevated METS-IR levels were significantly associated with an increased risk of OA. After adjusting for potential confounders, METS-IR was independently associated with OA (odds ratio (OR) = 1.026, 95% confidence interval (CI) = 1.020–1.033, *P* <.0001). Quartile analysis revealed that individuals in the highest METS-IR quartile had a significantly higher risk of OA compared to those in the lowest quartile (OR = 2.068, 95% CI = 1.617–2.644, *P* <.0001). Nonlinear analysis indicated a threshold effect, with METS-IR levels exceeding 42.153 significantly increasing OA risk (OR = 1.031, 95% CI = 1.022–1.040, *P* <.0001). ROC analysis showed that METS-IR had moderate predictive ability, with an AUC of 0.829 after full adjustment. Elevated METS-IR levels are significantly associated with increased OA risk, with a nonlinear relationship identified. METS-IR may serve as a potential predictor for OA, providing new insights into the “metabolic phenotype” hypothesis of OA and offering a basis for early screening and intervention. However, due to the cross-sectional design and reliance on self-reported OA diagnosis, causality cannot be established, and further longitudinal studies are warranted.

## 1. Introduction

Osteoarthritis (OA) is a chronic, progressive disease characterized by degenerative changes in the articular cartilage, with key clinical manifestations including joint pain, stiffness, and functional impairment.^[1]^ As the most prevalent joint disorder worldwide, OA is not only a leading cause of disability among middle-aged and elderly populations but also significantly reduces patients’ quality of life and imposes a substantial economic burden on healthcare systems.^[2,3]^ Traditionally, OA development has been closely associated with factors such as sex, genetic predisposition, obesity, abnormal bone density, joint malalignment, and muscle strength imbalance.^[4]^ However, emerging evidence suggests that metabolic abnormalities may play a crucial role in both the onset and progression of OA.^[5]^ Obesity contributes to OA not only as a mechanical risk factor but also by accelerating joint degeneration through metabolic mechanisms, including insulin resistance, adipokine imbalance, and chronic low-grade inflammation.^[6,7]^ Existing evidence indicates that female patients with knee OA exhibited higher the metabolic score for insulin resistance (METS-IR) levels than the national baseline, and that elevated METS-IR was significantly correlated with adipokine disorder and inflammatory activity.^[8]^ Other studies have suggested that metabolic syndrome (MetS), hypertension, and hyperglycemia were positively associated with knee OA.^[9]^ In addition, systematic reviews have highlighted that MetS is a multisystem disease linked not only to cardiovascular disorders but also to musculoskeletal impairments, reinforcing the systemic nature of OA as a metabolic phenotype.^[10]^ Collectively, these findings suggest that OA is not merely a mechanical joint disorder but a complex systemic condition closely linked to metabolic status. Therefore, a deeper understanding of the relationship between metabolic abnormalities and OA will not only enhance our knowledge of the disease’s pathophysiology but may also provide new insights for early prevention and targeted therapeutic interventions.

METS-IR is an emerging, comprehensive metabolic assessment index designed to quantitatively evaluate the degree of risk for insulin resistance and metabolic abnormalities. Insulin resistance is a pathological condition in which the body’s peripheral tissues become less responsive to insulin, resulting in a reduced efficiency of insulin-mediated glucose uptake and utilization, ultimately leading to hyperglycemia and hyperinsulinemia.^[11]^ As a core pathophysiological factor in type 2 diabetes mellitus and cardiovascular disease, insulin resistance is closely linked to various metabolic disorders, including obesity and MetS.^[12,13]^ Compared to traditional methods of assessing insulin resistance (e.g., HOMA-IR), METS-IR, as a non-insulin-dependent metabolic index, offers a more comprehensive reflection of an individual’s metabolic status by integrating several key indicators such as fasting blood glucose, triglycerides, HDL cholesterol, and body mass index (BMI). Existing studies have demonstrated that METS-IR possesses good sensitivity and specificity in predicting MetS, type 2 diabetes, and cardiovascular diseases.^[14–16]^ Furthermore, the calculation of METS-IR is straightforward and requires only routine blood test indicators, making it both clinically practical and valuable for research purposes. However, despite its widespread recognition in the field of metabolic diseases, research on the association between METS-IR and other chronic conditions, such as OA, remains limited. Therefore, further investigation into the potential relationship between METS-IR and OA could not only broaden the application of METS-IR but also provide new epidemiological evidence regarding the pathological link between metabolic abnormalities and OA.

Based on the research background outlined above, this study aims to systematically analyze the correlation between METS-IR and OA using the National Health and Nutrition Examination Survey (NHANES 2011–2018) database. Additionally, it will further evaluate the predictive efficacy of METS-IR in assessing the risk of OA and its potential pathogenic role in the development of the condition.

## 2. Materials and methods

### 2.1. Study design and population

**Figure 1.**
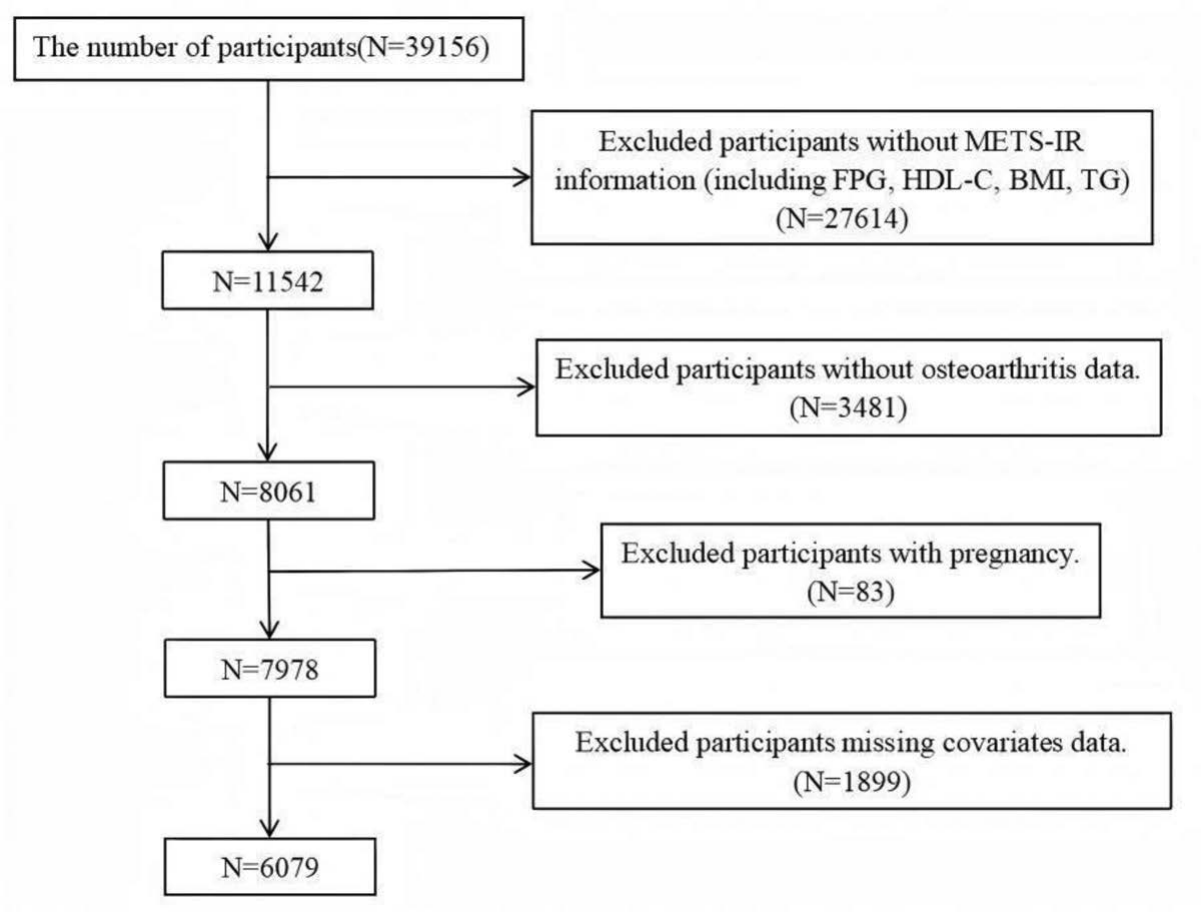
Baseline characteristics of participants.

This study utilized data from the NHANES 2011 to 2018 database to investigate the association between OA and the METS-IR in the U.S. adult population. NHANES (http://www.cdc.gov/nchs/nhanes.htm), a nationally representative cross-sectional health survey conducted by the Centers for Disease Control and Prevention, systematically collects comprehensive health data through interviews, physical examinations, laboratory tests, and imaging studies. The survey employs a complex, multi-stage probability sampling design, with biennial data collection cycles to ensure the reliability and representativeness of the national sample. The NHANES protocol has been reviewed and approved by the Research Ethics Review Board of the National Center for Health Statistics, and all adult participants provided written informed consent prior to their inclusion. The study was conducted in accordance with the ethical principles outlined in the Declaration of Helsinki. Further details regarding the Centers for Disease Control and Prevention and National Center for Health Statistics institutional review boards are available at (https://www.cdc.gov/nchs/nhanes/about/erb.html). From the NHANES 2011 to 2018 database, we initially identified 6079 participants aged 20 years or older (Fig. 1). Exclusion criteria included: missing METS-IR data, absence of OA-related data, pregnancy, and incomplete data on key covariates. A complete-case analysis was adopted. Individuals with missing values for the METS-IR index, OA, or any covariate were excluded from the final analysis. No statistical imputation was performed.

### 2.2. Assessment of osteoarthritis

The NHANES Medical Conditions Questionnaire, part of the “Medical Conditions” module, systematically collects data on arthritis. Participants were first asked, “Has a doctor or healthcare professional ever told you that you have arthritis?” Those who answered “yes” were further questioned: “What type of arthritis specifically?” Based on their responses, participants were classified into 3 groups: the OA group (individuals diagnosed with OA only), the other types of arthritis group (excluded from the analysis), and the no arthritis group. Physician-diagnosed OA based on self-reported data is a widely accepted and commonly used method in epidemiological studies. Although this approach ensures feasibility in large-scale population surveys, it may introduce information bias due to recall inaccuracy or misclassification. Nevertheless, self-reported physician diagnosis has been validated in prior NHANES-based studies and is considered reasonably reliable for estimating OA prevalence at the population level.^[17]^

### 2.3. Measurement of METS-IR index

In this study, METS-IR was treated as the exposure variable. The formula for METS-IR is as follows:

METS-IR = [Ln (2 × fasting blood glucose (mg/dL)) + fasting triglycerides (mg/dL)] × body mass index (kg/m^2^)]/ [Ln (high-density lipoprotein cholesterol (mg/dL))].^[15]^

Fasting blood glucose and triglyceride measurements were obtained using an automated biochemical analyzer with enzymatic methods. Specifically, serum triglyceride concentrations were determined using a Roche Cobas 6000 chemistry analyzer and a Roche Modular P system. Body mass index (BMI) was calculated by dividing weight (kg) by the square of height (m), with data on weight and height obtained from the “Body Measurements” screening data module in the NHANES database.

### 2.4. Covariates

Several potential confounders were included as covariates in the analysis of the association between METS-IR and OA. These covariates included sociodemographic variables such as age (classified into 3 groups: 20–49, 50–69, and ≥70 years), gender, and ethnicity; lifestyle factors including alcohol consumption (categorized as ≥4–5 drinks/day and <4–5 drinks/day) and smoking status (categorized as <100 cigarettes and ≥100 cigarettes in their entire life); and health status indicators such as the presence of diabetes, hypertension, and cancer.

### 2.5. Statistical analysis

To account for the complex sampling design of NHANES, appropriate weighting methods were applied in all statistical analyses to adjust for clustering and stratification effects. The normality of continuous variables was assessed using the Kolmogorov–Smirnov test. Categorical variables were analyzed using the chi-square test and expressed as percentages (%). Continuous variables were presented as mean ± standard deviation for normally distributed data or as median (interquartile range, IQR) for non-normally distributed data. For between-group comparisons, the Student *t* test was used for normally distributed variables, while the Mann–Whitney *U* test was applied for non-normally distributed variables. Multivariate logistic regression models were constructed to evaluate the association between METS-IR levels and OA, with results expressed as odds ratios (ORs) and their corresponding 95% confidence intervals (CIs). Four stepwise-adjusted models were developed: Model 1 (unadjusted crude model); Model 2 (adjusted for basic sociodemographic factors, including age, gender, and race); Model 3 (further adjusted for lifestyle factors, such as alcohol consumption and smoking status); and Model 4 (fully adjusted for all covariates, including age, gender, race, alcohol consumption, smoking status, diabetes, hypertension, and cancer). To investigate potential nonlinear relationships, a smoothed curve-fitting analysis was performed based on Model 4, and threshold effects were analyzed to identify critical values in the association between METS-IR and OA. Receiver operating characteristic (ROC) curve analysis was conducted to evaluate the predictive ability of METS-IR for OA risk by calculating the area under the curve (AUC) and its 95% CI for each of the 4 models. All statistical analyses were performed using EmpowerStats (www.empowerstats.com, X&Y Solutions, Inc., Boston) and R software (www.R-project.org, The R Foundation). A two-sided *P*-value <.05 was considered statistically significant.

## 3. Results

### 3.1. Baseline characteristics

A total of 6079 participants were included in this study, with 882 (14.5%) diagnosed with OA and 5197 (85.5%) without OA. Since OA diagnosis in NHANES relied on self-reported physician diagnosis, potential misclassification bias cannot be excluded. Table 1 presents the baseline characteristics of the study population. Significant differences were observed between the OA and non-OA groups in terms of gender, age, race, smoking status, and the prevalence of diabetes, hypertension, and cancer (*P* <.0001). However, no statistically significant difference was found between the 2 groups in alcohol consumption (*P* = .06).

**Table 1:** Characteristics of participants based on OA status, weighted.

Characteristic	Non-OA group (N = 5197)	OA group (N = 882)	*P*-value
Age (yr)	44.00 ± 16.12	60.80 ± 12.61	<.0001
20–49 yr (%)	64.15	17.26	
50–69 yr (%)	28.44	56.42	
≥70 yr (%)	7.41	26.32	
Gender (%)			
Male	55.81	37.26	<.0001
Female	44.19	62.74	
Ethnicity (%)			
Mexican American	9.58	3.32	<.0001
Other Hispanic	6.82	2.45	
Non-Hispanic White	65.53	83.18	
Non-Hispanic Black	9.96	5.42	
Other Ethnicities	8.11	5.63	
Alcohol use (%)			
≥4–5 drinks/d	14.98	18.14	.06
<4–5 drinks/d	85.02	81.86	
Smoking (%)			
≥100 cigarettes	44.85	56.97	<.0001
<100 cigarettes	55.15	43.03	
Diabetes, n (%)			
Yes	7.86	17.63	<.0001
No	92.14	82.37	
Hypertension (%)			
Yes	26.00	55.78	<.0001
No	74.00	44.22	
Cancer (%)			
Yes	7.04	21.56	<.0001
No	92.96	78.44	
METS-IR	42.23 ± 12.35	45.92 ± 14.10	<.0001
Q1 [17.25, 33.84] (N = 1520)	27.47	19.82	
Q2 [33.84, 41.14] (N = 1519)	24.56	23.48	
Q3 [41.14, 49.73] (N = 1520)	25.08	22.84	
Q4 [49.73, 131.37] (N = 1520)	22.89	33.86	

Mean ± SD for continuous variables: the *P*-value was calculated by the weighted linear regression model; (%) for categorical variables: the *P*-value was calculated by the weighted χ^2^ test.

METS-IR index = metabolic score for insulin resistance index, OA = osteoarthritis, Q1–Q4 = quartile 1–quartile 4.

### 3.2. Association between METS-IR and osteoarthritis

Table 2 presents the results of the association between METS-IR levels and OA. Univariate logistic regression analysis demonstrated a significant positive association between METS-IR and OA across all models: Model 1 (OR = 1.022, 95% CI = 1.017–1.028, *P* <.0001), Model 2 (OR = 1.032, 95% CI = 1.026–1.038, *P* <.0001), Model 3 (OR = 1.032, 95% CI = 1.025–1.038, *P* <.0001), and Model 4 (OR = 1.026, 95% CI = 1.020–1.033, *P* <.0001).

**Table 2:** Association between METS-IR index with osteoarthritis, weighted.

Characteristic	OR (95% CI), *P*-value			
	Model 1	Model 2	Model 3	Model 4
METS-IR	1.022 (1.017, 1.028) <.0001	1.032 (1.026, 1.038) <.0001	1.032 (1.025, 1.038) <.0001	1.026 (1.020, 1.033) <.0001
Quartile 1 (N = 1520)	Reference	Reference	Reference	Reference
Quartile 2 (N = 1519)	1.209 (0.970, 1.508) .09	1.165 (0.912, 1.488) .22	1.158 (0.906, 1.481) .24	1.071 (0.835, 1.373) .58
Quartile 3 (N = 1520)	1.353 (1.089, 1.680) .006	1.446 (1.134, 1.843) .003	1.423 (1.115, 1.815) .005	1.238 (0.964, 1.590) .09
Quartile 4(N = 1520)	2.078 (1.694, 2.550) <.0001	2.612 (2.071, 3.294) <.0001	2.548 (2.020, 3.216) <.0001	2.068 (1.617, 2.644) <.0001
*P* for tend	<.0001	<.0001	<.0001	<.0001

Model 1: crude model; Model 2: adjusted for gender, age and ethnicity; Model 3: adjusted for gender, age, ethnicity, smoking and alcohol use; Model 4: adjusted for gender, age, ethnicity, smoking, alcohol use, diabetes, hypertension and cancer.

CI = confidence interval, OR = odds ratio.

Further analysis stratified by METS-IR quartiles revealed that participants in the highest quartile (Q4) had a significantly elevated risk of OA compared to those in the lowest quartile (Q1) across all adjusted models: Model 2 (OR = 2.612, 95% CI = 2.071–3.294, *P* <.0001; *P* for trend <.0001), Model 3 (OR = 2.548, 95% CI = 2.020–3.216, *P* <.0001; *P* for trend <.0001), and Model 4 (OR = 2.068, 95% CI = 1.617–2.644, *P* <.0001; *P* for trend <.0001).

**Figure 2.**
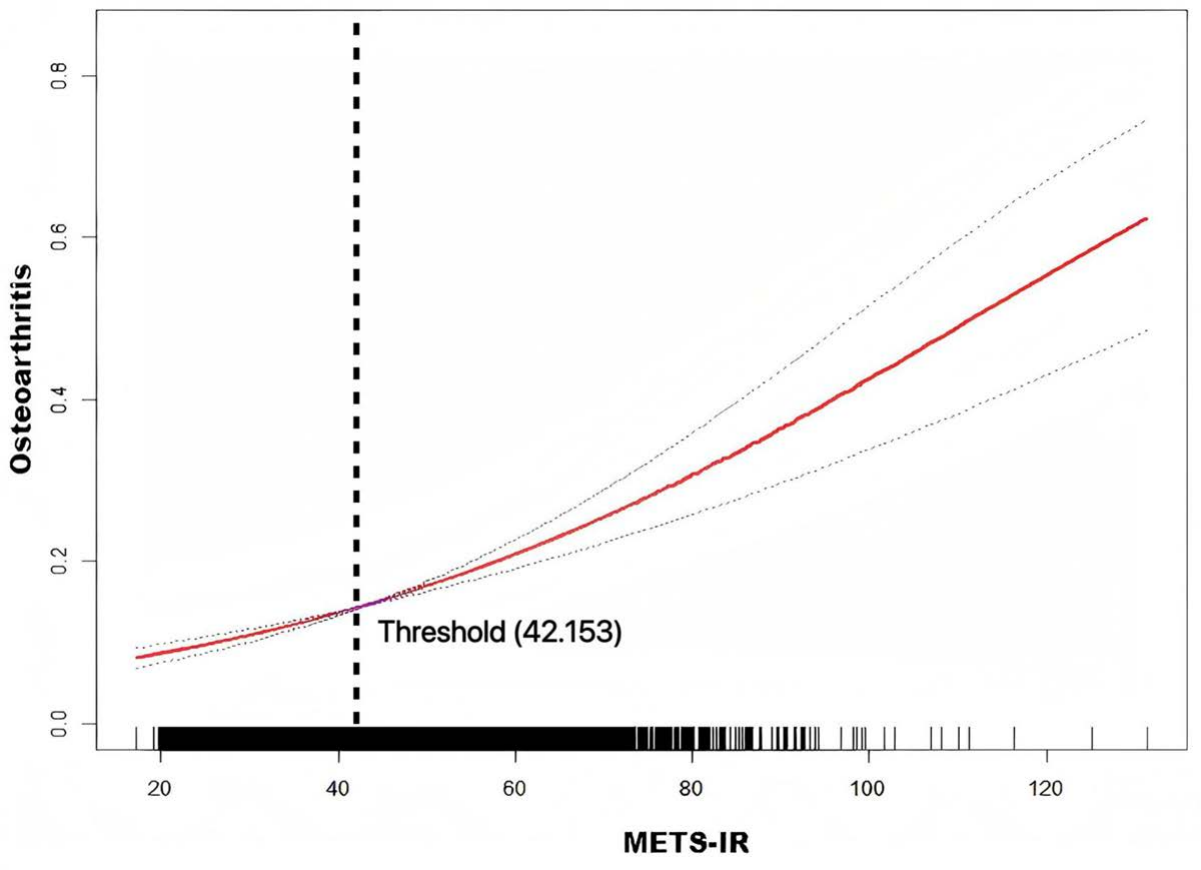
Smooth curve fitting for the relationship between METS-IR index and osteoarthritis risk. The area between 2 blue dotted line is on behalf of a 95% CI. The red dotted line suggests the positive linear relationship between METS-IR index and osteoarthritis risk. CI = confidence interval, METS-IR = the metabolic score for insulin resistance.

Smoothed curve-fitting analyses revealed a nonlinear relationship between METS-IR levels and OA risk after adjusting for all covariates (Fig. 2). Threshold effect analysis identified a critical METS-IR value of 42.153. When METS-IR exceeded this threshold, the risk of OA increased significantly with higher METS-IR levels (OR = 1.031, 95% CI = 1.022–1.040, *P* <.0001).

### 3.3. Subgroup analysis

Subgroup analyses were conducted to assess the potential effect of METS-IR on OA risk after adjusting for all covariates (Table 3). The analysis revealed that the effect of METS-IR on OA risk remained significantly positive in subgroups stratified by gender, age, smoking status, alcohol consumption, diabetes, and hypertension. Interaction tests indicated no significant interactions in the subgroups of sex, age, race, alcohol consumption, diabetes, or cancer (*P* >.05). Notably, significant interactions were observed in the smoking and hypertension subgroups (*P* <.05).

**Table 3:** Subgroup analysis for the association between METS-IR index and osteoarthritis.

Variables	OR	95% CI	*P* for interaction
Gender			
Male	1.023	1.013, 1.033	.86
Female	1.030	1.021, 1.038	
Age			
20–49 yr	1.027	1.016, 1.039	.45
50–69 yr	1.020	1.011, 1.030	
≥70 yr	1.023	1.008, 1.039	
Ethnicity			
Mexican American	1.027	1.000, 1.054	.98
Other Hispanic	1.024	0.999, 1.050	
Non-Hispanic White	1.026	1.017, 1.035	
Non-Hispanic Black	1.025	1.011, 1.040	
Other ethnicities	1.022	0.999, 1.046	
Alcohol use			
≥4–5 drinks/d	1.017	1.002, 1.031	.79
<4–5 drinks/d	1.029	1.021, 1.036	
Smoking			
≥100 cigarettes	1.021	1.012, 1.030	.03
<100 cigarettes	1.033	1.023, 1.043	
Diabetes			
Yes	1.029	1.015, 1.043	.31
No	1.025	1.017, 1.032	
Hypertension			
Yes	1.028	1.020, 1.037	.03
No	1.020	1.009, 1.030	
Cancer			
Yes	1.024	1.006, 1.042	.97
No	1.027	1.019, 1.034	

All models were adjusted for gender, age, ethnicity, smoking, alcohol use, diabetes, hypertension and cancer.

CI = confidence interval, OR = odds ratio.

### 3.4. Stratified analysis by age

To thoroughly address the potential effect modification by age, we conducted a stratified analysis wherein the fully adjusted model (Model 4) was run separately within each age group. The results are presented in Table 4. When METS-IR was treated as a continuous variable, the positive association with OA was consistent and statistically significant across all 3 age strata. When analyzed by quartiles, a notable pattern emerged: the strength of the association between high METS-IR (Quartile 4 vs Quartile 1) and OA was strongest in the 20 to 49 years age group (OR = 2.647, 95% CI: 1.600–4.380, *P* <.0001), moderate in the 50 to 69 years group (OR = 1.749, 95% CI: 1.224–2.498, *P* = .002), and attenuated yet still significant in the ≥70 years group (OR = 1.704, 95% CI: 1.045–2.780, *P* = .03).

**Table 4:** Stratified analysis by age.

Characteristic	N	OR (95% CI)	*P*-value
METS-IR (continuous variable)			
20–49 yr	3293	1.027 (1.016, 1.039)	<.0001
50–69 yr	1966	1.020 (1.011, 1.030)	<.0001
≥70 yr	820	1.023 (1.008, 1.039)	.002
METS-IR (quartile variable)			
20–49 yr			
Quartile 1	935	Reference	
Quartile 2	760	1.273 (0.713, 2.273)	.41
Quartile 3	764	1.713 (0.984, 2.981)	.05
Quartile 4	834	2.647 (1.600, 4.380)	<.0001
50–69 yr			
Quartile 1	390	Reference	
Quartile 2	527	1.030 (0.722, 1.468)	.87
Quartile 3	536	1.007 (0.703, 1.444)	.96
Quartile 4	513	1.749 (1.224, 2.498)	.002
≥70 yr			
Quartile 1	195	Reference	
Quartile 2	232	0.985 (0.635, 1.527)	.94
Quartile 3	220	1.365 (0.868, 2.147)	.17
Quartile 4	173	1.704 (1.045, 2.780)	.03

All models were adjusted for gender, ethnicity, smoking, alcohol use, diabetes, hypertension and cancer.

CI = confidence interval, OR = odds ratio.

### 3.5. ROC curve analysis

**Figure 3.**
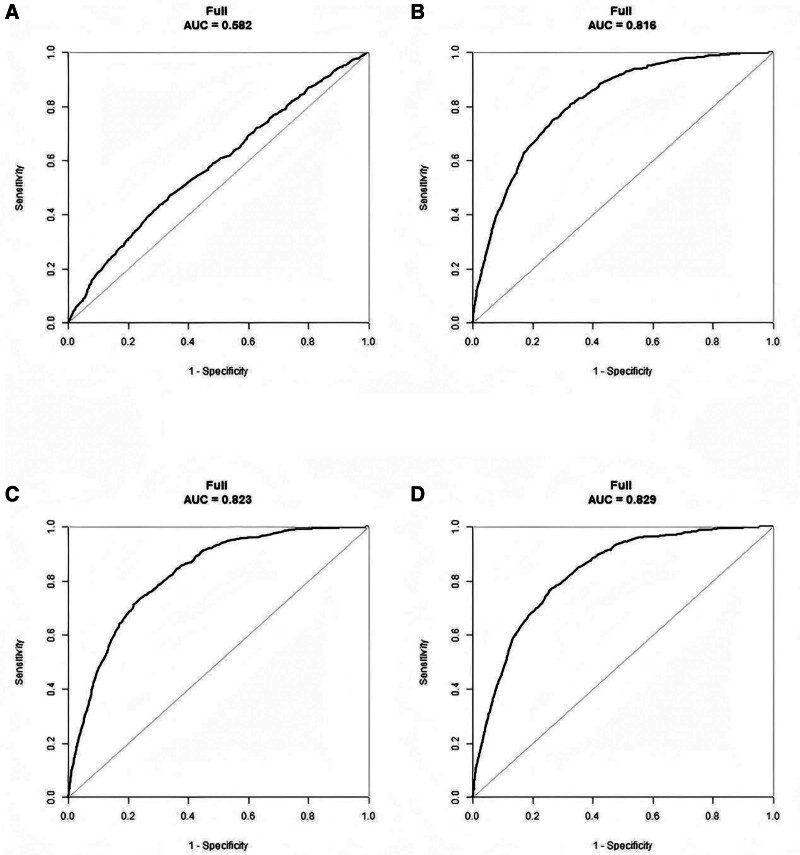
ROC curve analysis for the predictive efficacy of METS-IR for OA risk. (A) Model 1: unadjusted model; (B) Model 2: adjusted for gender, age and ethnicity; (C) Model 3: adjusted for gender, age, ethnicity, smoking and alcohol use; (D) Model 4: adjusted for gender, age, ethnicity, smoking, alcohol use, diabetes, hypertension and cancer. METS-IR = the metabolic score for insulin resistance, OA = osteoarthritis, ROC = receiver operating characteristic.

To evaluate the predictive efficacy of METS-IR for OA risk, we conducted a ROC curve analysis in 4 models (Fig. 3). In the unadjusted model, the AUC for METS-IR (continuous variable) predicting OA was 0.582 (95% CI: 0.561–0.602, *P* <.0001). As the models were progressively adjusted, the predictive efficacy improved significantly: Model 2 (AUC: 0.816, 95% CI: 0.802–0.830, *P* <.0001), Model 3 (AUC: 0.823, 95% CI: 0.809–0.836, *P* <.0001) and Model 4 (AUC: 0.829, 95% CI: 0.816–0.842, *P* <.0001). Model 4 demonstrated the best predictive efficacy, with a sensitivity of 76.4% and a specificity of 74.6%.

## 4. Discussion

This study explored the association between METS-IR and OA using data from the NHANES database. The results showed that elevated METS-IR levels were significantly and positively associated with the risk of OA. After adjusting for potential confounders, METS-IR remained an independent risk factor for OA. Additionally, quartile analysis revealed that individuals in the highest METS-IR quartile exhibited a significantly higher risk of OA compared to those in the lowest quartile, suggesting a dose-dependent effect of METS-IR. Further smoothed curve fitting analysis indicated a nonlinear positive correlation between METS-IR levels and OA risk, with a significant increase in risk when METS-IR exceeded a critical threshold value of 42.153. The robustness of this association is further underscored by our stratified analysis, which confirmed that the positive relationship between METS-IR and OA remained consistent and statistically significant across all age groups. Subgroup analyses revealed that the effect of METS-IR on OA risk was more pronounced in individuals who smoked or had hypertension, suggesting potential interactions between these factors and METS-IR. ROC curve analysis demonstrated that METS-IR had a strong predictive ability for OA, with Model 4 providing the best predictive efficacy.

Our findings support the “metabolic phenotype” hypothesis of OA, which suggests that metabolic abnormalities, rather than mechanical factors alone, play a significant role in the pathogenesis of OA. Previous studies have indicated that insulin resistance, a key component of metabolic abnormalities, is closely linked to various metabolic disorders, including type 2 diabetes and cardiovascular diseases.^[18–20]^ This study extends these findings by demonstrating that METS-IR, a comprehensive metabolic assessment index, is also significantly associated with OA risk. Elevated METS-IR levels reflect multiple metabolic abnormalities, including insulin resistance, lipid dysregulation, and obesity, all of which contribute to the development and progression of OA.

Regarding the mechanisms underlying the relationship between METS-IR and OA, current research suggests several possible pathways. First, chronic low-grade inflammation is considered a critical link between insulin resistance and OA. Insulin resistance can lead to elevated levels of systemic inflammation and activation of proinflammatory cytokines, which in turn can stimulate the major intracellular inflammatory pathways, such as NF-κB and JNK, leading to synovial inflammation, cartilage matrix degradation, and chondrocyte apoptosis.^[21,22]^ These inflammatory mediators can accelerate OA progression by promoting joint degradation.^[23–25]^ Additionally, recent reviews and experimental studies indicate that insulin/insulin-like growth factor signaling exerts a protective effect on chondrocytes by inhibiting apoptosis and promoting matrix synthesis and cell proliferation, whereas insulin resistance or metabolic imbalance weakens these protective mechanisms.^[5,26]^ Another important factor is lipotoxicity, which is commonly associated with insulin resistance. Insulin resistance typically leads to lipid metabolism disorders, resulting in elevated plasma levels of free fatty acids, which can directly affect chondrocytes, promoting oxidative stress and inflammation, and accelerating cartilage degradation.^[5,27]^ Furthermore, adipose tissue, as an active endocrine organ, secretes various adipokines, such as leptin and adiponectin, which have been implicated in OA progression. Specifically, leptin levels are significantly elevated in the synovial fluid of OA patients and promote articular cartilage degradation via the JAK/STAT signaling pathway. Insulin resistance can further enhance the pro-inflammatory effects of leptin, exacerbating joint damage.^[28]^

A notable finding in this study was the identification of a significant nonlinear relationship between METS-IR and OA. The smoothed curve-fitting analysis revealed that when METS-IR levels were below 42.153, the association with OA was weak. However, once METS-IR exceeded this threshold, the risk of OA increased significantly (*P* <.0001). This nonlinear trend may be related to the biological effects of insulin resistance. In the early stages of insulin resistance, the body may maintain glucose homeostasis through compensatory insulin hypersecretion, potentially mitigating some of its adverse effects on joint tissues.^[29,30]^ However, when METS-IR levels persistently rise and exceed the compensatory capacity, the resulting hyperinsulinemia and metabolic disturbances may lead to more severe damage to joint tissues, accelerating the progression of OA.^[31]^ Additionally, the hyperglycemic state that typically accompanies insulin resistance can contribute to OA through mechanisms such as the deposition of advanced glycation end products (AGEs), oxidative stress, and inflammatory responses.^[32,33]^ This suggests that METS-IR may serve as a useful clinical threshold for early detection and intervention in OA.

Subgroup analyses revealed significant interactions between METS-IR and smoking and hypertension. The association between METS-IR and OA was stronger in individuals with hypertension, possibly due to vascular dysfunction, increased oxidative stress, and a chronic inflammatory state induced by hypertension. Hypertension can impair the microvascular network of subchondral bone, reducing nutrient supply to cartilage and accelerating OA progression.^[34,35]^ Additionally, hypertensive patients often have comorbid MetS, which may exacerbate the inflammatory response and enhance the detrimental effects of insulin resistance on joint health. Similarly, the association between METS-IR and OA was more pronounced in smokers, likely due to the systemic inflammation and oxidative stress caused by smoking. Smoking promotes the release of pro-inflammatory cytokines, induces cartilage degradation, and may further impair insulin sensitivity, accelerating OA progression.^[36–38]^ These findings suggest that smoking and hypertension may act synergistically to increase the risk of OA in individuals with elevated METS-IR.

While this study provides important epidemiologic evidence regarding the association between METS-IR and OA, there are some limitations. First, because of a cross-sectional design, the association remains unclear whether a high level of METS-IR precedes development of OA or whether immobility related to OA contributes to elevation in METS-IR levels. Second, the diagnosis of OA was based on self-reported physician diagnoses rather than radiographic or clinical confirmation. We reiterate that this method, while necessary for large-scale epidemiology, is susceptible to misclassification bias. It is likely that participants with undiagnosed or mild OA were included in the non-OA group, which would typically bias the results toward the null and lead to an underestimation of the true effect size. Third, we adopted a complete-case analysis approach to handle missing data, excluding participants with any missing information. Although the proportion of missingness was low, this method assumes data are missing at random. If this assumption is violated, it could introduce selection bias. Furthermore, although we adjusted for several confounders, residual confounding due to factors such as dietary habits, physical activity levels, and other variables that may influence insulin resistance and OA was not accounted for in this analysis. Lastly, the NHANES data is primarily based on the U.S. population, and the generalizability of these findings to other populations requires further validation.

In conclusion, this study provides novel evidence of the significant association between METS-IR and OA. Elevated METS-IR levels are positively correlated with increased OA risk, with a nonlinear relationship observed. These findings support the “metabolic phenotype” hypothesis of OA and suggest that METS-IR may be a valuable predictor for assessing OA risk. However, given the cross-sectional design, our study should be regarded as hypothesis-generating rather than predictive, highlighting the need for prospective cohort studies to validate these associations. Future studies should focus on elucidating the biological mechanisms linking METS-IR and OA and validating its predictive value in longitudinal studies to facilitate early intervention strategies for OA.

## 5. Conclusions

Elevated METS-IR levels are significantly associated with increased OA risk, with a nonlinear relationship identified, suggestive of a potential value for risk stratification in future prospective studies, providing novel epidemiological support for the “metabolic phenotype” hypothesis of OA. These findings suggest that METS-IR warrants further investigation in longitudinal studies to elucidate its potential value for risk stratification and early screening.

## Acknowledgments

We thank all the participants of the study.

## Author contributions

**Conceptualization:** Fangjun Xiao.

**Data curation:** Junpeng Qiu, Muyuan Hou.

**Formal analysis:** Junpeng Qiu.

**Methodology:** Junpeng Qiu, Muyuan Hou, Junxing Yang, Fangjun Xiao.

**Resources:** Yaoxin Ao.

**Software:** Junpeng Qiu, Muyuan Hou.

**Validation:** Jiangfeng Lv, Yaoxin Ao, Yifei Liufu.

**Writing – original draft:** Junpeng Qiu, Muyuan Hou.

**Writing – review & editing:** Jiangfeng Lv, Yaoxin Ao, Yifei Liufu, Junxing Yang, Fangjun Xiao.
